# Mixed Matrix Membrane for Cr(VI) Adsorption and Reduction: Joining Click Chemistry and MOFs

**DOI:** 10.1021/acsomega.6c00237

**Published:** 2026-05-12

**Authors:** Stefano Torresi, Stefano Elli, Javier Martí-Rujas, Nagore Gabilondo, Arantxa Eceiza

**Affiliations:** † Materials + Technologies’ Research Group, Department of Chemical and Environmental Engineering, Faculty of Engineering of Gipuzkoa, 16402 University of the Basque Country UPV/EHU, Plaza Europa 1, Donostia 20018, Spain; ‡ Department of Chemistry, Materials and Chemical Engineering “Giulio Natta”, 18981 Politecnico di Milano, Via Luigi Mancinelli 7, Milan 20131, Italy

## Abstract

In this work, a maleimide-bearing biobased polyurethane is made to react with a metal–organic framework (MOF) containing thiol pendant groups (Zr-MSA), to produce a functional mixed matrix membrane (MMM). The membrane is synthesized following an innovative method in which Zr-MSA acts as a cross-linker through UV-promoted thiol-Michael addition while providing the ability to trap Cr­(VI) and reduce it to Cr­(III). By means of a comprehensive study of the surface morphology and adsorption kinetics, the adsorption mechanism is elucidated. The homogeneous distribution of the MOF allows for a high cross-linking degree and adsorption ability, resulting in an MMM with good mechanical properties and filtering capacity. Using the thiol-maleimide click reaction, this new strategy permits easy incorporation of MOFs into a membrane without affecting their functionality, leveraging them as active cross-linkers.

## Graphical abstract

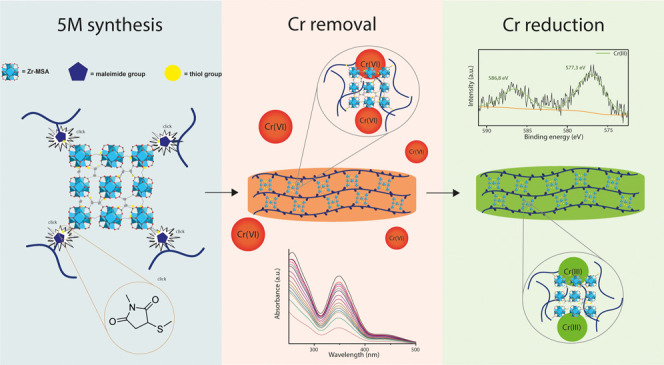

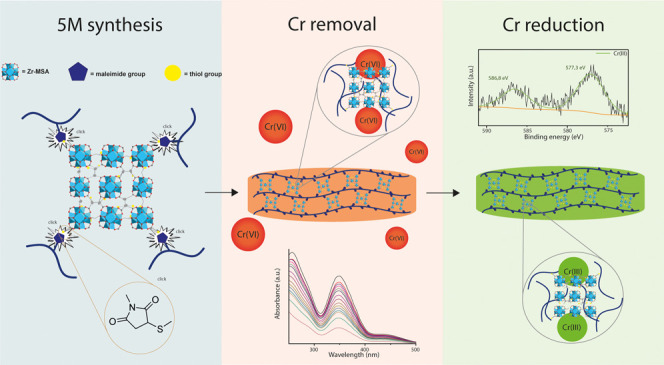

## 1 Introduction

In recent years, MOFs have been intensively studied for their versatility, easy and quantitative synthesis, and high stability in different environments. Between the various characteristics, the large surface-to-volume ratio and the great number of periodically distributed metal active sites result in size-tunable open cavities where molecules can be stored. A wide range of functionalized Zirconium-based MOFs can be synthesized thanks to the vast selection of commercially available dicarboxylic acid ligands. These MOFs find application in various fields such as catalysis, gas separation, and ion removal.^1–5^ Depending on the MOF’s application, they can be obtained as large single crystals, microcrystalline powders, thin films, or gels.^6–8^ Between the most studied and used MOFs, the UiO66 type stands out due to its easy and scalable synthesis but also thanks to its resistance in acidic environments, which make them interesting for application in industrial conditions.^9,10^

The main drawback of using MOF powders as a filtering agent lies in their recovery after the adsorption process. In fact, due to the small size of such a crystalline structure, it may be awkward to recover it.^11,12^ To solve this problem, the crystals could be immobilized on a polymeric substrate, using two different approaches: pre- or postsynthesis functionalization processes. The pre-synthesis processes deal with the dispersion of the MOF in the polymeric solution and its subsequent production as substrate through different techniques (electrospinning, wet spinning, solvent casting)^13–15^ to fabricate mixed-matrix membranes (MMM). For example, Cohen et al. managed to synthesize MMMs by solvent casting, with a high content of different MOFs (up to 90 wt %), maintaining good membrane flexibility.^16,17^ Instead, Hmadeh et al. obtained antibacterial electrospun mats using polyvinyl chloride and postmetalated UiO-66­(COOH)_2_ and ZIF-8.^18^ Between the great varieties of postsynthesis functionalization, some consist in chemical reaction to create nucleation sites for MOF growth.^19,20^ In this context, a widely used strategy is the in situ solvothermal growth, where the porous support is immersed in the precursor solution to obtain polycrystalline membranes via heterogeneous nucleation.^21^ A similar technique is the layer-by-layer growth, which allows for a precise control over the membrane structure, enabling the fine-tuning of the thickness by the number of growth cycles,^22,23^ while others perform chemical or physical deposition (carboxymethylation, grafting of carboxylate pendant functionalities, ionic liquid welding, or atomic layer deposition).^24–28^ The incorporation of MOFs in different matrices concerns several compatibility issues, typical of all the composites, which are by no means trivial. Indeed, MMMs often present interfacial adhesion problems resulting in weak domains and voids that greatly affect their final performances. This leads to poor membrane selectivity, which results in low adsorption or separation efficiency. Raising the MOF content results in a significant drawback, specifically agglomeration, given by the low affinity of polymers to inorganic fillers, which makes it very challenging to obtain a fine dispersion of the crystals.^29–31^ A direct consequence of that is the limitation of the membrane surface area and the resulting structural weakening of the entire structure given by rigid agglomeration in the polymeric matrix.^32^ Instead, when the substrate is just decorated with MOFs, detachment problems often appear coupled with a hard and time-consuming functionalization process.^33,34^ To solve these problems, the post-synthetic modification (PSM) of MOFs was proved to be a very effective solution for the production of MOF-polymer hybrid materials.^35,36^ Even if the introduction of chemical reactive groups can result in a reduced adsorption capacity, this technique can be used to functionalize the MOFs with specific chemical groups designed to react with the polymer chains, enabling their grafting onto the polymer.^37^ Among the different reactions available, click chemistry stands as a powerful tool, which is already widely used for modification and cross-linking of different types of polymeric materials, due to orthogonality, high yield, and absence of byproducts.^38–44^ A novel approach would immobilize the MOFs into the membrane through a click reaction, using them as cross-linkers. In this way, precise spatial arrangement and strong matrix–filler interaction are provided, thus resulting in a better dispersion of the MOFs and improved membrane performances.^45,46^ Just a few studies explored the use of UV-promoted thiol–ene click reaction as a method for obtaining cross-linked MMM, using different catalysts.^47,48^

In the present work, applying the principles of click chemistry and using a highly stable, readily producible Zr-MSA MOF as a cross-linking agent, a novel MMM was obtained. As the matrix, a biobased polyurethane bearing pendant maleimide groups (PU-MAL) was used, while the filler was a Zr-based MOF containing thiol groups, Zr-MSA, synthesized following the work of Yang et al.^2^ Thereby, through UV-promoted thiol-maleimide click reaction, without the use of any catalyst, the MOF-maleimide MMM (5M) was obtained. The preparation strategy proposed herein allows for an efficient immobilization of MOFs, which act as active fillers and cross-linkers simultaneously. A uniform distribution of the crystals all over the matrix was achieved, resulting in an MMM behaving as a solid-like gel. The adsorption capacity of 5M for the removal of Cr­(VI) from aqueous solutions was tested, and the data were fitted using different kinetic models. Moreover, it was confirmed that the concomitant reduction of Cr­(VI) to less toxic Cr­(III). This work provides a facile and scalable production method for a functional MMM suitable to be used in the removal of contaminants from aqueous media.

## 2 Materials and Methods

### 2.1 Materials

The polyurethane (PU) with maleimide pendant groups (PU-MAL) was synthesized using a biobased poly­(butylene sebacate)­diol (PBSD) derived from castor oil as soft segment (3505 g mol^–1^; 32 mg KOH g^–1^ OH index, ASTM D 4274-88 Test Method A). PBSD biobased carbon content was 72% according to the ASTM-D6866–12 (0.98) Method B (AMS) standard procedure.^49^ The hard segment was constituted by 1,6-hexamethylene diisocyanate (HDI, 168 g mol^–1^), kindly provided by Covestro AG, and N-(2,3-dihydroxypropyl) maleimide (DHPM) was synthesized as explained in section 2.2. Zirconium chloride (ZrCl_4,_ 233 g mol^–1^), mercaptosuccinic acid (MSA, 150.15 g mol^–1^), formic acid (FA, 46.03 g mol^–1^), exo-3,6-epoxy-1,2,3,6-tetrahydrophthalic anhydride (Furan-A, 166.13 g mol^–1^), and (±)-3-amino-1,2-propanediol (APO, 91.11 g mol^–1^) and dithiothreitol (DTT, 154.2 g mol^–1^) were purchased from Sigma-Aldrich. 2,3-dimercaptosuccinic acid (DMSA, 182.22 g mol^–1^) was purchased from TCI chemicals. *N*,*N*-dimethylformamide (DMF) and chloroform (CHCl3) were purchased from Scharlau. Ethanol (EtOH) and tetrahydrofuran (THF) were purchased from Sigma-Aldrich.

### 2.2 Synthesis of *N*-(2,3-Dihydroxypropyl)­maleimide

The DHPM chain extender provided pendant maleimide groups to the PU and was synthesized following our previous work.^50^ Briefly, 10 g (0.06 mol) of Furan-A and 5.65 g (0.062 mol) of APO were dissolved in 45 mL of EtOH and refluxed at 85 °C for 24 h. The white solid obtained, *N*-(2,3-dihydroxy-propyl)-10-oxa-4-aza-tricyclo­[5.2.1.02,6]-dec-8-ene-3,5-dione (DHPM-A, 232 g mol^–1^), was deprotected through the retro-Diels–Alder reaction by refluxing at 125 °C for 7 h in THF. The final product *N*-(2,3-dihydroxypropyl) maleimide (DHPM, 174 g mol^–1^) was collected through vacuum filtration, washed with petroleum ether, and dried under vacuum at 60 °C for 24 h. ^1^H NMR and ^13^C NMR and ^15^N of DHPM are shown in Figure S1.

### 2.3 Synthesis of PU-MAL

PU-MAL was synthesized using a two-step or prepolymer bulk polymerization method, following a previous protocol.^51^ First, PBSD (14.9 g, 4.25 mmol) was melted in a two-necked round flask purged with N_2,_ and HDI (2.86 g, 17 mmol) was added and mechanically stirred at 90 rpm and made to react at 90 °C for 2 h. Then, DHPM chain extender (2.22 g, 12.7 mmol) was added to the prepolymer, and mixed at 150 rpm for 10 min. The viscous liquid was transferred to a mold and pressed at 100 °C and 50 bar for 10 h, to complete the polymerization. No catalyst was used. Films of 90 mm long, 90 mm wide, and 1.4 mm thick were produced. The polyol:diisocyanate:chain extender (PBSD:HDI:DHPM) molar ratio used was 1:4:3. The hard segment percentage was calculated as 25 wt %. PU-MAL was cut into small pieces and dissolved at 20 wt % in DMF:CHCl_3_ (2:1 v/v), by stirring at 40 °C to obtain a pale yellow, homogeneous solution.

### 2.4 Synthesis of Zr-MSA MOF

Zr-MSA MOF was synthesized following the procedure of Yang et al.^2^ Briefly, 2.33 g (10 mmol) of ZrCl_4_, 1.5 g (10 mmol) of MSA, and 1.6 mL of FA were dissolved in 20 mL of water. After sonication for 5 min, the mixture was transferred to a closed vial and put in an oven at 80 °C for 2 h. Then, the solid was washed with H_2_O (twice) and EtOH (three times) and let in ethanol 4 h. Finally, it was dried at 80 °C for 24 h in vacuum.

For comparative purposes, a MOF containing 2,3-dimercaptosuccinic acid as ligand (Zr-DMSA) was also synthesized. To do so, 2.33 g (10 mmol) of ZrCl_4_, 1.82 g (10 mmol) of DMSA, together with 0.68 mL of FA, were dissolved in 20 mL of water, sonicated for 5 min, and finally transferred to a closed vial and heated at 90 °C for 12 h. Then, the obtained MOF was washed with H_2_O (twice) and ethanol (once), soaked in ethanol for 4 h, and dried at 80 °C for 24 h in a vacuum. Both MOFs were stored under N_2_ until their use. A SEM image of Zr-MSA is shown in Figure S2.

### 2.5 5M Production Process

Zr-MSA MOF was used as a cross-linking agent of PU-MAL by means of a thiol-Michael addition reaction. In our supposition, among all the thiol groups of the MOF, some would be involved in the thiol-maleimide reaction, whereas some would remain available for the chemisorption of Cr­(VI). Several variables would play important roles in this theoretical assumption, such as the MOF agglomeration, the oxidation degree of thiol groups, the stereochemistry and spatial arrangement of thiol and maleimide groups, among others. Therefore, it was decided to disperse the MOFs in one of the two solvents used to dissolve the polyurethane. It was found that Zr-MSA was better dispersed in DMF than in CHCl_3_. Following a general procedure for the dispersion and mixing of MOF,^15^ Zr-MSA was ground using a mortar and a pestle, and the resulting powder was ultrasonicated in DMF for 20 min, obtaining a homogeneous solution with a concentration of 300 mg mL^–1^. Then, 1 mL of PU-MAL solution (20 wt %) was put in a vial under vigorous stirring, and 0.1 mL of MOF dispersion (300 mg mL^–1^) was added dropwise. The dispersion was stirred for another 5 min and subsequently sonicated for 5 min. Finally, the vial was irradiated with UV light at 365 nm for 2 h. The resulting membrane is shown in Figure S3. The amount of Zr-MSA solution added was chosen as the lowest quantity of MOF necessary to obtain a self-standing membrane, in order to be sure that the vast majority of the crystals would participate in the cross-linking and not simply be dispersed in the matrix. A blank probe was also produced using DTT as a cross-linking agent of the PU-MAL. Here, 0.1 mL of a DTT solution of (30 mg mL^–1^) was dissolved in DMF and then added dropwise to 1 mL of PU-MAL solution (20 wt %). In this case, the solution gelled instantaneously. It is noteworthy to say that an attempt was made to obtain a gel using Zr-DMSA as a cross-linker, but it was not possible to obtain a cross-linked network. This could be due to the high steric hindrance of the DMSA, which prevents a proper organization of the thiol group, thus impeding the click reaction.

## 3 Results and Discussion

The 5M membranes were obtained through an easy process, where the PU-MAL solution and the MOF dispersion were mixed together, followed by irradiation with UV light, as described before. From the results of powder X-ray diffraction (PXRD) analysis in Figure 1A, the MOF presented a very high crystallinity, with a structure perfectly matching the one presented in the literature.^2^ Some characteristic peaks of Zr-MSA could be recognized in the diffractogram of 5M, confirming the effective incorporation of the MOF into the MMM. Furthermore, it was also checked that the structure of Zr-MSA would not change during the production process of 5M, as can be seen in Figure S4. FTIR analysis was performed to assess the occurrence of the click reaction between the pendant maleimide of PU-MAL and the thiol groups of Zr-MSA. In fact, as demonstrated by other studies,^52^ the characteristic peaks of the maleimide can be recognized at 696 and 829 cm^–1^. As can be seen from the comparison with the pristine PU-MAL (Figure 1B), in the 5M spectrum, both are strongly reduced, confirming that the maleimide had partly reacted with the thiol.^50^ The presence of residual DMF and water was evidenced from the peaks at 1395 cm^–1^ (N–CH_3_ bending) and 3370 cm^–1^ (O–H stretching), respectively.

**Figure 1:**
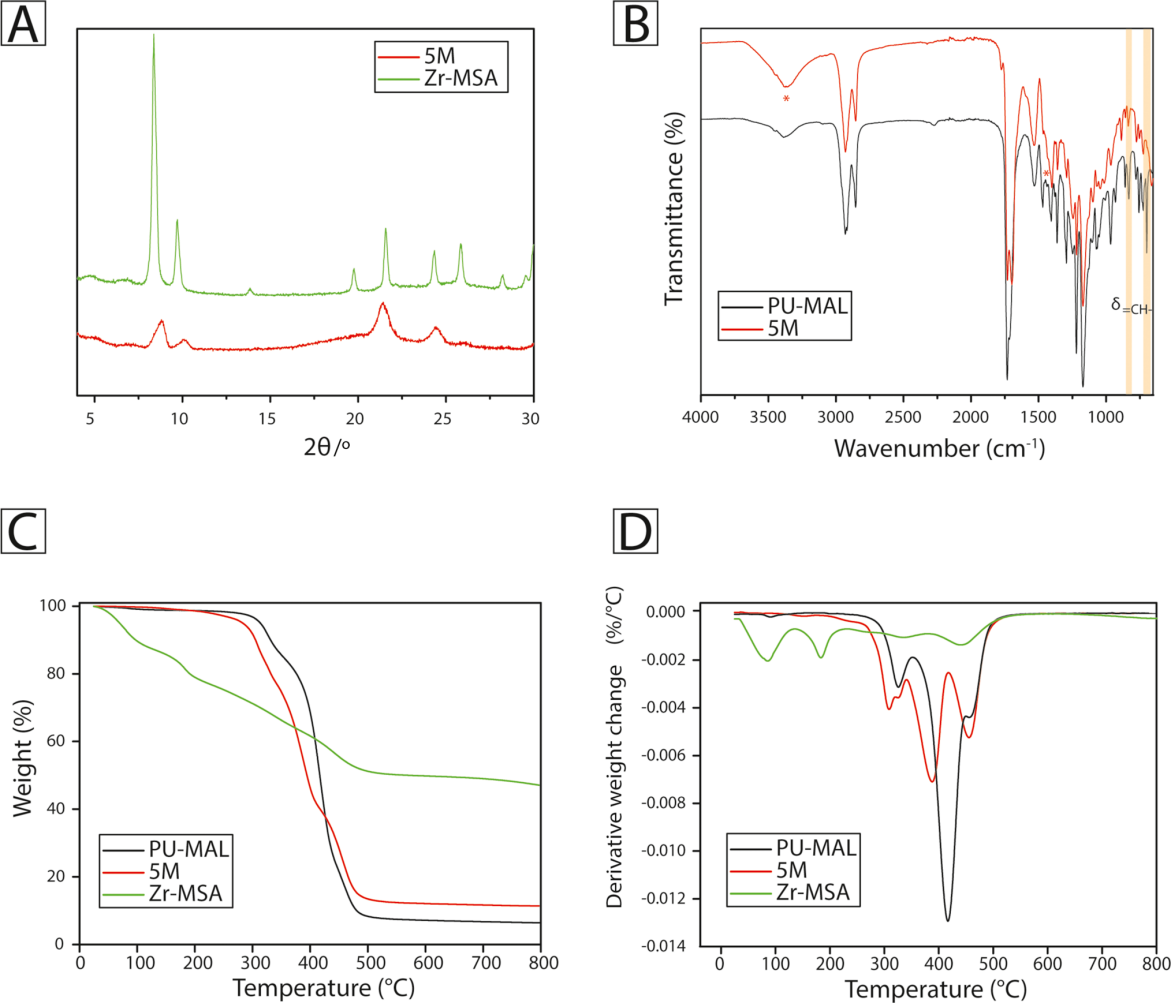
(A) PXRD diffractograms of 5M and Zr-MSA, (B) FTIR spectra of PU-MAL and 5M, (C) weight evolution with temperature, and (D) its derivative of PU-MAL, 5M and Zr-MSA.

Thermal degradation of 5M was analyzed by TGA (Figure 1C,D), and relevant differences were observed compared to the pristine polymer. Indeed, 5M shows a first weight loss around 306 °C, not present in PU-MAL curves, given by the initial degradation of the not-cross-linked MOF.^53^ Around 324 °C, in both compositions, the peak typical of castor-oil-based segmented polyurethanes can be appreciated, corresponding to the degradation of the urethane bonds.^54,55^ However, 5M shows a second weight loss around 387 °C, given by the degradation of the urethane groups attached to the chain extender involved in the cross-linking. The temperature shift could be explained by the stabilizing effect imparted by the high thermal stability of the MOF used as cross-linkers.^56^ Finally, the char residue of 5M was almost twice that of PU-MAL, due to the contribution of both MOFs and cross-linking, favoring the formation of a higher amount of char layer.

The surface morphology and internal microstructure of 5M were analyzed to investigate the distribution of the MOF in the matrix. From the AFM cross-section images (Figure 2A,B) the homogeneous distribution of the MOF throughout the membrane thickness could be confirmed. Besides, the high roughness of the surface was also appreciated, which was related to the cross-linking degree since the higher the cross-linking density, the rougher the surface of the material.^57^ SEM images of the membranes were taken before and after the Cr­(VI) adsorption process. As shown in Figure 2C, the characteristic geometric pattern of Zr-MSA was easily identified in the matrix, and the MOF’s crystals seemed uniformly distributed within the sample. As shown in Figure S5, there were no noticeable differences between the mixed matrix membranes before and after the adsorption process, at least morphologically, indicating the good stability of 5M in water. However, from a macroscopic point of view, the different steps of the adsorption process were recognizable from the color variations of the membrane that changed from pale yellow, the proper color of the PU-MAL, to red first and to green later, as illustrated in Figure S6, which denoted the adsorption and reduction of Cr­(VI) to Cr­(III), respectively. The adsorption of chromium was also corroborated by EDX analysis. Prior to the Cr­(VI) adsorption, the presence of sulfur, S, on the sample was confirmed, and its distribution was mapped over the matrix (Figure S7). As it could be observed, a quite uniform distribution was detected, with a few denser spots, which were attributed to MOF aggregates. Thereafter, the EDX analysis on the 5M after the adsorption of Cr­(VI) was performed. As shown in Figure 2D,E, the distribution of both Cr and S matched almost perfectly, confirming the adsorption ability of the Zr-MSA.

**Figure 2:**
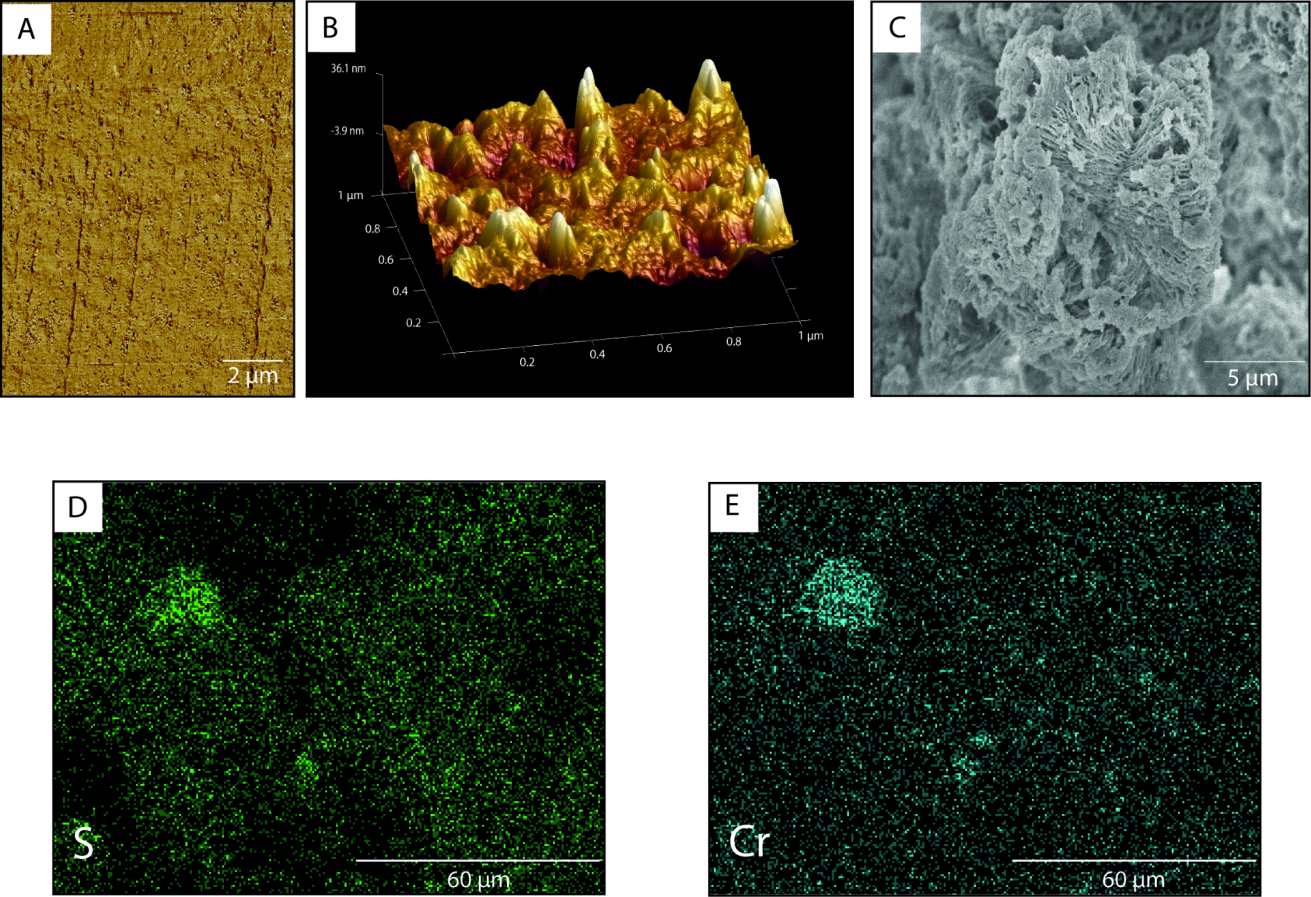
(A) Cross-section AFM image of 5M, (B) 3D topographic AFM image of 5M, (C) SEM image of 5M before Cr­(VI) adsorption, and (D) EDX images of S and (E) EDX images of Cr distribution, both images taken after the adsorption process.

Integrity of 5M was measured by means of oscillatory frequency sweep rheological test (Figure S8) and from the rheological measurements, network structural parameters such as average mesh size (ξ), cross-linking density (ne), and average molecular weight between neighboring cross-links (Mc) were calculated to be 3.03 nm, 144.43 mol m^–3,^ and 1381 kg mol^–1^. The results showed a very small mesh size, which can be attributed to the high polymer concentration of the PU-MAL solution used (20 wt %), as well as the high molar concentration of clickable groups and the excellent reactivity of the Zr-MSA, which results in a densely cross-linked material. To confirm such assumptions, a water uptake test was carried out on 5M samples. Indeed, the water uptake was very small, reaching a mean value of 17%, confirming the difficult penetration of H_2_O molecules inside the polymer network due to the high cross-linking density and small porosity.^58^

To understand the adsorption mechanism and capacity, the pore diameter and distribution were characterized. The BET curves obtained for 5M (Figure 3A) show a material with a surface area of 8.123 m^2^ g^–1^, which is considerably lower than the reported surface area of around 512 m^2^ g^–1^ for free Zr-MSA in the literature.^2,59^ The isotherm presented by the MMM can be labeled as a type-III isotherm, which is characteristic of mesoporous or nonporous materials, where the adsorbed molecules form clusters around specific sites, in this case, the Zr-MSA crystals present on the surface of 5M. The thin H1 type hysteresis, ranging between 0.3 and 0.95 P/P0, can be assigned to the ink-bottle pore network.^60^ Besides, the pore size distribution (Figure 3A, inset) was centered on 17.45 Å. Those findings present 5M as a nonporous membrane. The water uptake and the adsorption kinetics results seem to corroborate this hypothesis. Once characterized, both the internal and surface morphologies of 5M, its capacity as a Cr adsorbent, were measured. In a typical experiment, the adsorbent concentration was set to 1000 mg L^–1^, that of the Cr­(VI) was 100 mg L^–1^, while pH and temperature were set to 6 and 25 °C, respectively. The adsorption was monitored using UV–vis spectroscopy, taking as reference the peak at 349 nm typical of the Cr­(VI) in water at pH 6. Fifteen aliquots were taken during 24 h. Figure 3B shows that the main adsorption step occurred within the first hour, where 30% of the Cr­(VI) was removed from the medium. The maximum adsorption capacity was assessed to be 70.2 mg g^–1^ after 24 h with a removal efficiency of 77%. It is worthy to note that the adsorption capacity values were calculated just considering the Zr-MSA of the 5M sample as the adsorbent. This assumption was made after assessing both through EDX analysis and UV–vis spectroscopy, that Zr-MSA was the only Cr­(VI) adsorbent in 5M. Indeed, the absorption spectra of Zr-MSA and the blank PU-MAL (Figure 3C,D) clearly support what was stated before. Higher Cr­(VI) concentrations were also tested, reaching the value of 360 and 2000 mg L^–1^ of adsorbent. As expected, the removal efficiency and the maximum adsorption capacity were sensibly higher, reaching after 24 h values of 93% and 180 mg g^–1^, respectively. This last experiment was performed during 48 h to see if any desorption occurred, finding out that no desorption occurred and, on the contrary, the adsorption continued (Figure S9).

**Figure 3:**
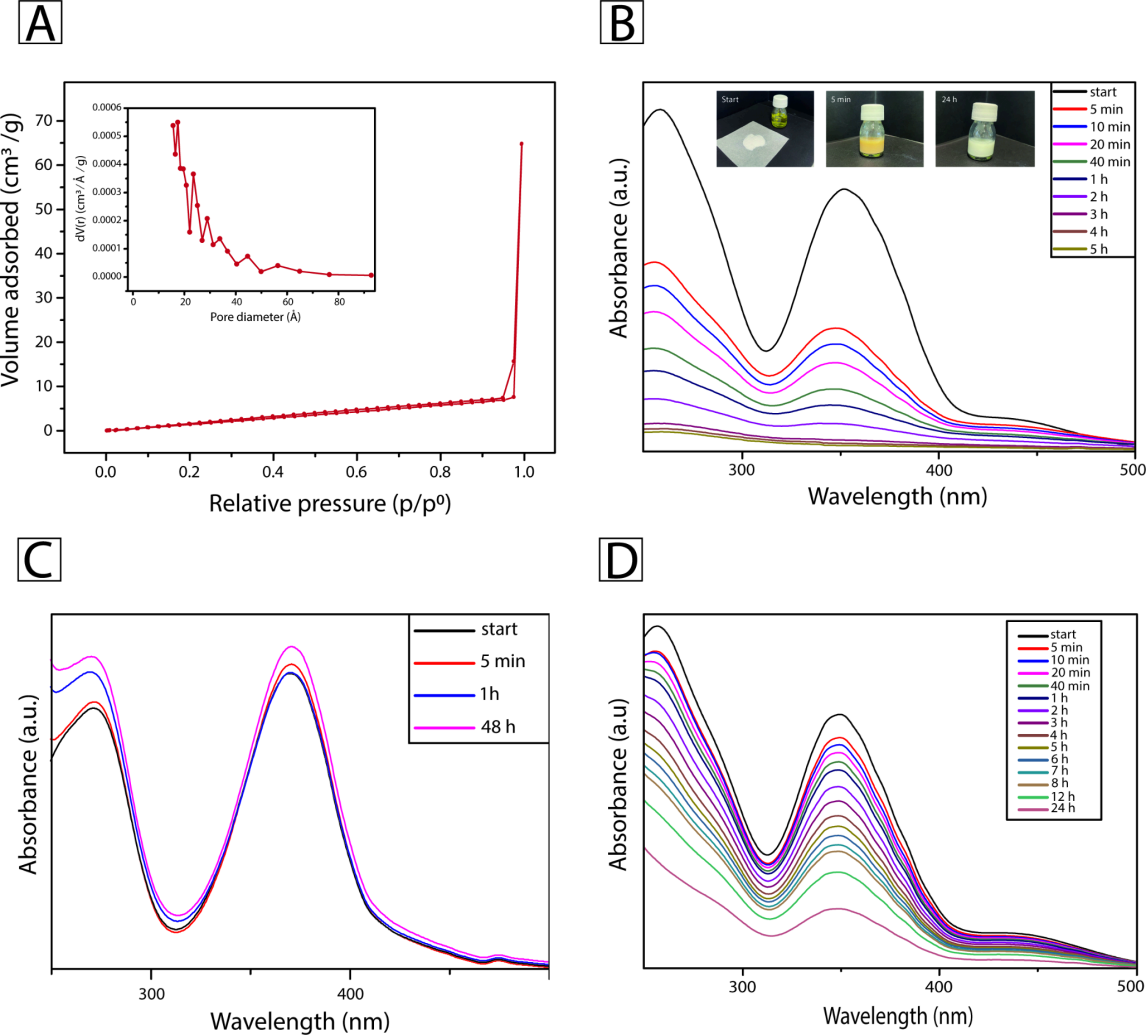
(A) BET isotherm plot of 5M, in the inset is shown the pore size distribution; UV–vis absorption spectra of the Cr­(VI) aqueous solution with (B) Zr-MSA; (C) PU-MAL and (D) 5M; recorded at different times. It can be clearly seen the strong decrease of the absorption peak of B and the negligible decrease of the absorption peak of C.

Considering that the Zr-MSA powder shows a very fast adsorption of Cr­(VI) (Figure S10) and the unreal possibility that the adsorbate diffusion in solution can be the limiting step, the most probable kinetics models to be used are the adsorption and diffusional ones. Furthermore, a nonporous structure was assumed for 5M with a very small pore size. From the previous considerations, it was deduced that the mechanism was initially governed by a fast adsorption process, assigned to the chemisorption of Cr­(VI) on the easily accessible MOFs on the surface. Once the adsorption sites available on the surface were saturated, the adsorption turned out to be governed by the diffusion process inside the mesh of the matrix.^61,62^ In Figure 4A, the two different kinetics can be recognized, one lasting approximately 20 min and another governing the rest of the experiment. Therefore, the data were initially fitted to the pseudo-second-order (PSO) model, as for the Zr-MSA in previous studies, and then to the Morris and Weber equation, the most appropriate for dense membranes, at 5M.^63^ The PSO has a very high *R* ^2^ value of 0.999 and an RMSE of 0.15, resulting in a very tight fitting of the model. The *k*_2_ constant was found to be 0.01 g mg^–1^ min^–1^, and the qe (17.54 mg g^–1^) is lower than the one calculated for the free Zr-MSA powder, considering that just a small amount of the total MOF added was present on the surface. The Weber-Morris model also fitted the remaining data quite well, with an *R* ^2^ of 0.98 and RMSE of 2.43. The *k*_p_ was found to be 1.74 mg g^–1^ min^–1/2^, and C equal to 8.3 mg g^–1^. Even if we believe that applying different models at different times was the most correct physical interpretation of the adsorption process, it makes the comparison with previous studies quite challenging, due to the tendency in the literature to fit all the data using just one model.

**Figure 4:**
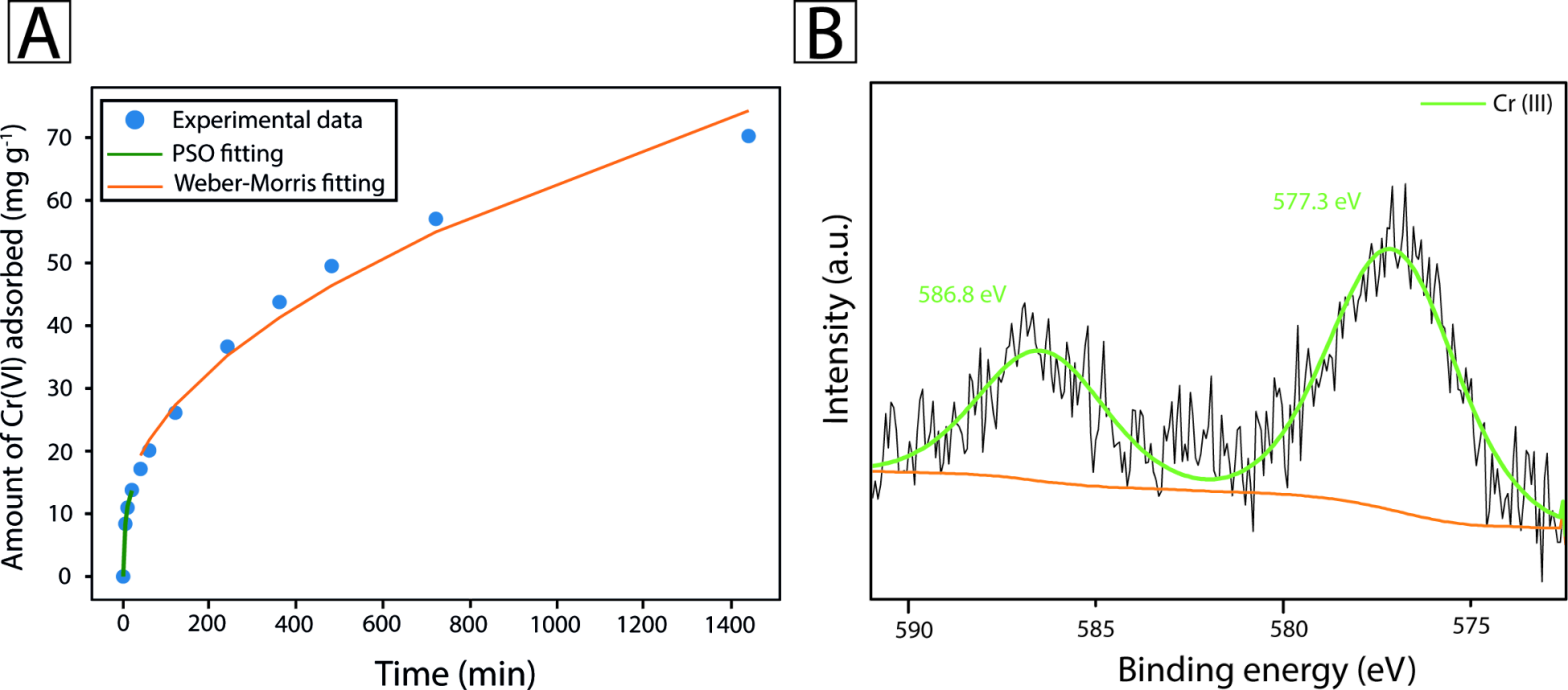
(A) Fitting of the absorption data of 5M, performed following during the first 20 min, the PSO model (green line) and after the Morris and Weber model (orange line) and B) XPS spectrum of 5M after absorption and reduction of Cr­(VI).

Finally, the effective reduction of Cr­(VI) to Cr­(III) was confirmed by means of XPS. Given the presented results, it is thought that the 5M removal and reduction of chromium mechanism starts from the initial chemisorption of Cr on the accessible MOFs available on the surface of 5M. Here, through a 3-step process, the Cr­(VI) is initially trapped in the frameworks of the MOF via the formation of a transition-metal–thiolate complex. Subsequently, it is reduced by the free thiol groups present on the ligand MSA, possibly passing through different intermediate oxidation states, obtaining the stable Cr­(III), which is finally retained inside the Zr-MSA cage by electrostatic interactions.^2,64^ This process could also be followed from the color changes, as explained before. However, the best way to probe the Cr reduction was to analyze the 5M by XPS. The Cr 2p spectrum of Cr­(VI) is characterized by a main peak around 579 eV and a smaller one around 589 eV, while Cr­(III) shows a characteristic peak around 577 eV and a secondary one around 587 eV.^2,65^ The spectra of 5M, as shown in Figure 4B, show just the characteristic peak of Cr­(III), confirming the effective reduction of the adsorbed Cr­(VI).

## 4 Conclusion

Here, for the first time, to the best of the authors’ knowledge, a thiol-maleimide reaction was used to cross-link a polymer using MOFs. A detailed study on the MMM composition, morphology, and porosity was conducted to shed light on the way the MOF is incorporated in the polymer matrix, concluding that 5M presented a homogeneous distribution of the MOF crystals, which were not simply embedded into the matrix, but chemically bonded to the polymer chain, thus gaining stability and avoiding problems of detachment. Furthermore, through the reduction of Cr­(VI) to Cr­(III), it was demonstrated that the MOFs' pores are still accessible and functional. The fast and easy synthesis route resulted in a MMM with good rheological properties, high adsorption capacity, and redox ability. These features, jointly with the reliable adsorption performance and the absence of desorption, make 5M promising for applications in the removal of contaminants from aqueous media. Finally, thanks to the uncountable MOFs containing or functionalized with clickable groups, the strategy used in this work paves the way for the synthesis of many other functional materials.

## Supplementary Material



## Notes

Techniques used to characterize the materials; NMR of the chain extender (S1), SEM images of the MOFs (S2), photos of the membrane (S3), PXRD of the MOFs (S4), morphology of 5M (S5), color change during Cr removal and reduction (S6), SEM-EDX of the pristine MOF (S7), rheological characterization of the membrane (S8), UV–vis spectra (S9), and kinetics of the absorption process (S10) (PDF)
