# Supplementary material for: Mixed Matrix Membrane for Cr(VI) Adsorption and Reduction: Joining Click Chemistry and MOFs
Source: ACS Omega. 2026 May 12;11(20):29808–16. doi: 10.1021/acsomega.6c00237 (PMC13216974; doi:10.1021/acsomega.6c00237)
Supplement: Supplementary file 1 [file ao6c00237_si_001.pdf]

Supporting Information

Mixed matrix membrane for Cr(VI) adsorption and reduction: joining click chemistry and MOFs

Stefano Torresi[a], Stefano Elli[b] Javier Martí-Rujas[b], Nagore Gabilondo[a] Arantxa Eceiza[a]**

[a.] Materials + Technologies' Research Group, Department of Chemical and Environmental Engineering, Faculty of Engineering of Gipuzkoa, University of the Basque Country UPV/EHU, Plaza Europa 1, Donostia 20018, Spain

[b.] Department of Chemistry, Materials and Chemical Engineering "Giulio Natta", Politecnico di Milano, Via Luigi Mancinelli 7, Milan 20131, Italy

1. Characterization

PXRD analysis was performed using a Bruker D2-Phaser diffractometer equipped with Cu radiation (λ=1.54184 Å) using Bragg Brentano geometry. The experiments were conducted at room temperature. Fourier-transform infrared spectroscopy (FTIR) spectra were recorded between 4000 and 650 cm^{-1}, performing 32 scans with a resolution of 4 cm^{-1} using a Nicolet Nexus spectrometer equipped with a MKII Golden Gate accessory (Specac) involving a diamond crystal at a nominal incidence angle of 45° and ZnSe lens. Thermogravimetric analysis (TGA) were performed using a TGA/DSC3+ Mettler Toledo equipment, heating the samples in a nitrogen

atmosphere from 25 to 800 °C, at a constant rate of 10 °C min^{-1}. The surface of the membranes was analysed with a Hitachi S-4800 scanning electron microscope (SEM), at 5 kV accelerating voltage. Morphological characterization and elemental composition were also analysed by scanning electron microscopy with energy dispersive X-ray analysis (SEM-EDX) using a Carl Zeiss EVO 40 equipped with an Oxford Instrument X-Max EDS detector. The conditions were set as 20 kV voltage, 100-200 pA current and 8.5 mm working distance, in high vacuum. The surface and cross-section morphology of 5M was characterized through Atomic force microscopy (AFM) using a Bruker Dimension ICON AFM, equipped with a Nanoscope VI controller. To that end, TESP-V2 type rectangular silicon tips with a nominal resonance frequency of 320 kHz, a cantilever spring constant of 42 N m^{-1} and a tip radius of 7 nm were employed. Viscoelastic properties of 5M were characterized in a Haake Viscotester iQ (Thermo Scientific), using a parallel plate geometry with a diameter of 35 mm (P35/Al adapter) and a working gap of 1 mm. Tests were performed in triplicate at 25 °C. First, in order to determine the linear viscoelastic region (LVR), amplitude tests were carried out, at a fixed frequency of 1Hz and a strain sweep of 0.1-100%. Frequency sweep tests were performed at a fixed strain in the LVR, from 0.1 to 50 Hz. The average mesh size (ξ, nm), the crosslinking density (ne, mol m^{-3}) and the average molecular weight between neighboring crosslinks (Mc, kg mol^{-1}) were determined by eq 1-3.

$$\xi = \left(\frac{G' \times N_A}{RT}\right)^{-1/3} \quad (1)$$

$$ne = \frac{Ge}{RT} \quad (2)$$

$$Mc = \frac{c\rho RT}{Ge} \quad (3)$$

where G′ is the storage modulus, N_A is the Avogadro constant ($6.022 \cdot 10^{23}$ mol^{-1}), R is the gas constant (8.314 J K^{-1} mol^{-1}), T is the temperature (293 K), Ge (Pa) is the storage modulus in the plateau region of the frequency sweep test, c is the polymer concentration (% w/v) and ρ is the

density of water at 293 K (998 kg m^{-3}). The water uptake was measured submerging the 5M samples in water at room temperature for 24 h and weighed after being wiped with a soft tissue. The water uptake was calculated as:

$$Water\ uptake\ (\%) = \frac{W_w - W_d}{W_d} \times 100$$

where Ww and Wd relate to the wet and dry weight values, respectively. The average pore diameter and specific surface area of 5M were determined from nitrogen adsorption-desorption isotherms at 77 K using the methods of Barrett-Joyner-Halenda (BJH) and Brunauer, Emmett and Teller (BET), respectively. Samples were characterized using Autosorb iQ (Quantachrome) after outgassing solid samples at 323 K for 24 h. The adsorption kinetics of Cr(VI) was followed using Ultraviolet-visible (UV–vis) spectroscopy using a Shimadzu UV-3600. Calibration curve was constructed from aqueous solutions of $K_2Cr_2O_7$ by diluting the initial batch solution of 2,000 mg L^{-1} to 20, 40, 80, 100, 200, 300, 400, 500 and 1,000 mg L^{-1} solutions. The reference peak of Cr(VI) was chosen at 349 nm. The pH of the solution was assessed to be equal to 6.

The adsorption behaviour of the free Zr-MSA powder was characterized using the same conditions used for 5M membranes, so the adsorbent concentration was set to 1,000 mg L^{-1} while that of the Cr(VI) was 100 mg L^{-1}.To perform the UV-vis analysis, just the supernatant of the decanted solution was analysed.

In a closed vial, 40 mg of 5M were added to 3 mL of Cr(VI) solution. The absorbance values were taken at 0, 5, 10, 20, 40, 60 minutes and at 2, 3, 4, 5, 6, 7, 8, 12 and 24 hours. The removal efficiency (R) (mg g^{-1}) and the equilibrium adsorption capacity (q_e) of 5M were calculated using the following equations:

$$R = \frac{C_0 - C_{24h}}{C_0} \times 100 \ (4)$$

$$q_e = \frac{(C_0 - C_e) \times V}{m} \quad (5)$$

where C_0, C_{24h} and C_e are the Cr(VI) concentration (mg L^{-1}) at the beginning, 24 h and equilibrium, respectively, while V and m are the volume of the Cr(VI) solutions (L) and the adsorbent mass (mg) for the adsorption experiment. Data fitting was performed using Python 3.10 software.

The equation describing the Pseudo Second Order (PSO) model was:

$$q_t = \frac{k_2 {q_e}^2 t}{1 + k_2 q_e t} \quad (6)$$

while the equation describing the Weber and Morris model was:

$$q_t = k_p t^{1/2} + C \quad (7)$$

where q_t is the amount of adsorbed Cr at time t (mg g^{-1}), k_2 is the rate constant of the PSO (mg g^{-1} min $^{-1}$), k_p is the interparticle diffusion constant (mg g^{-1} $min^{-1/2}$) and C is a constant (mg g^{-1}).

The value of R^2 and RMSE were calculated using the following equations:

$$R^2 = \frac{SS_{res}}{SS_{tot}} \quad (8)$$

$$RMSE = \sqrt{\frac{\sum_{i=1}^{N} (P_i - O_i)^2}{N}} \quad (9)$$

where SS_{res} is the sum of residual errors while SS_{tot} is the total sum of errors, P_i is the predicted value of the ith observation, O_i is the observed value of the ith observation and N is the size of the sample.

For X-ray photoelectron spectroscopy (XPS) analysis, the samples, after the adsorption process, were dried at 45 °C under vacuum for 24 hours. Then, they were grinded and analysed using a SPECS system equipped with a Phoibos 150 1D-DLD analyser with a monochromatic radiation of Al K (1486.7 eV).

1. NMR characterization of DHPM.

In figure S1 are shown the labelled ^{1}H NMR and ^{13}C NMR of DHPM.

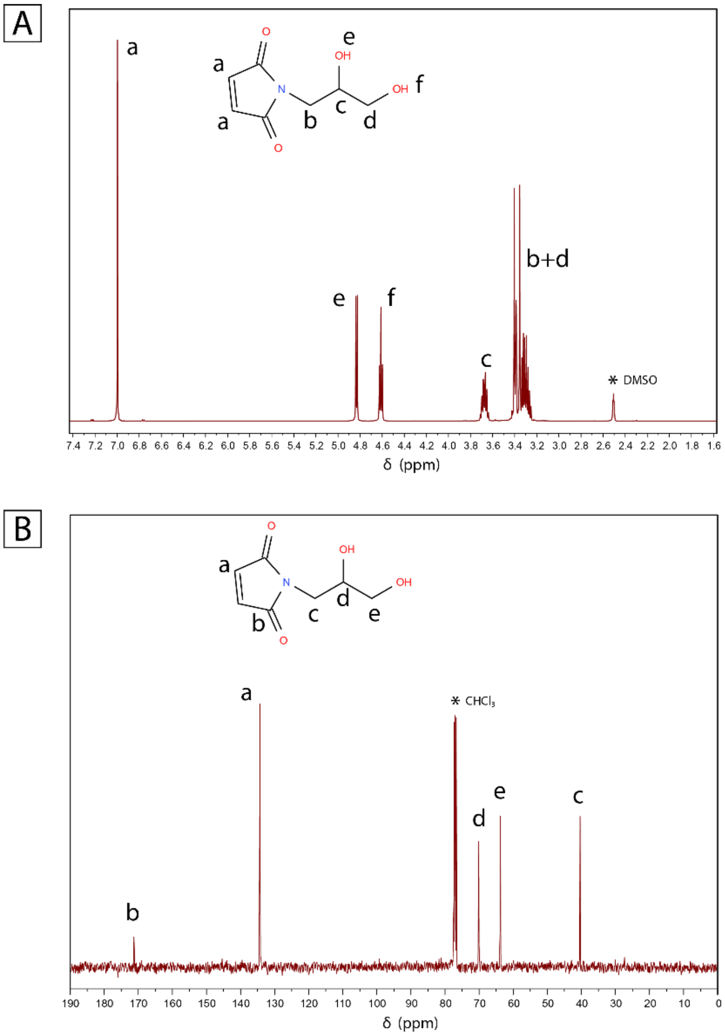

Figure S1 A) ^{1}H NMR and B) ^{13}C NMR of the synthesized DHPM.

2. MOFs' morphology

A SEM image at higher magnification of MOFs, showing their morphology.

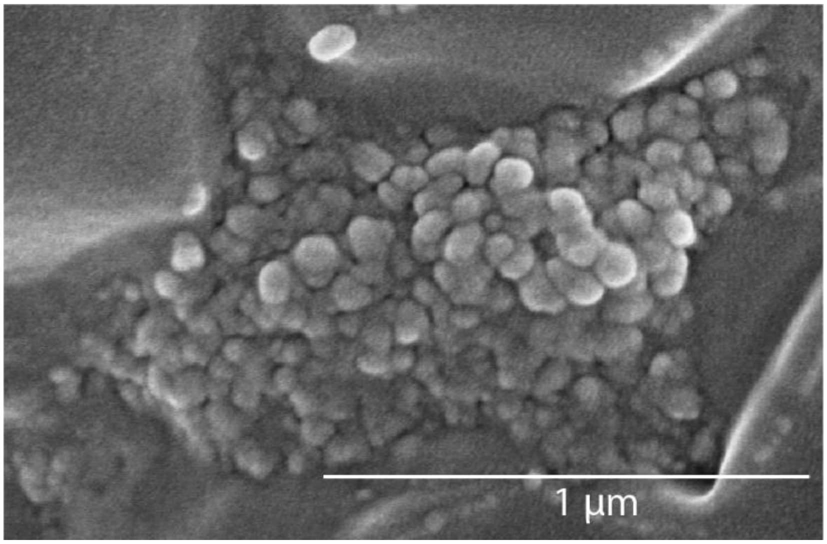

Figure S2 SEM of Zr-MSA

3. Photos of the membrane.

In Figure S1 is shown the membrane as synthesized. The good handleability can be appreciated in the photos on the right.

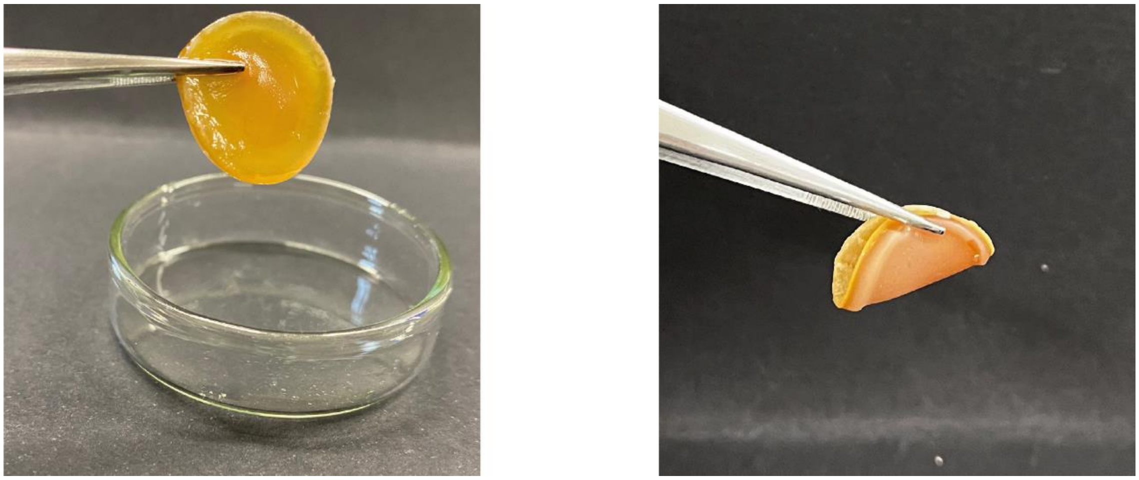

Figure S3 Photos of 5M as synthesized.

4. PXRD analysis

The PXRD analysis was performed in order to confirm the successful synthesis of the Zr-MSA. The crystalline structure of Zr-MSA was checked after each step of the 5M production process, to be sure that no changes occurred (Figure S2).

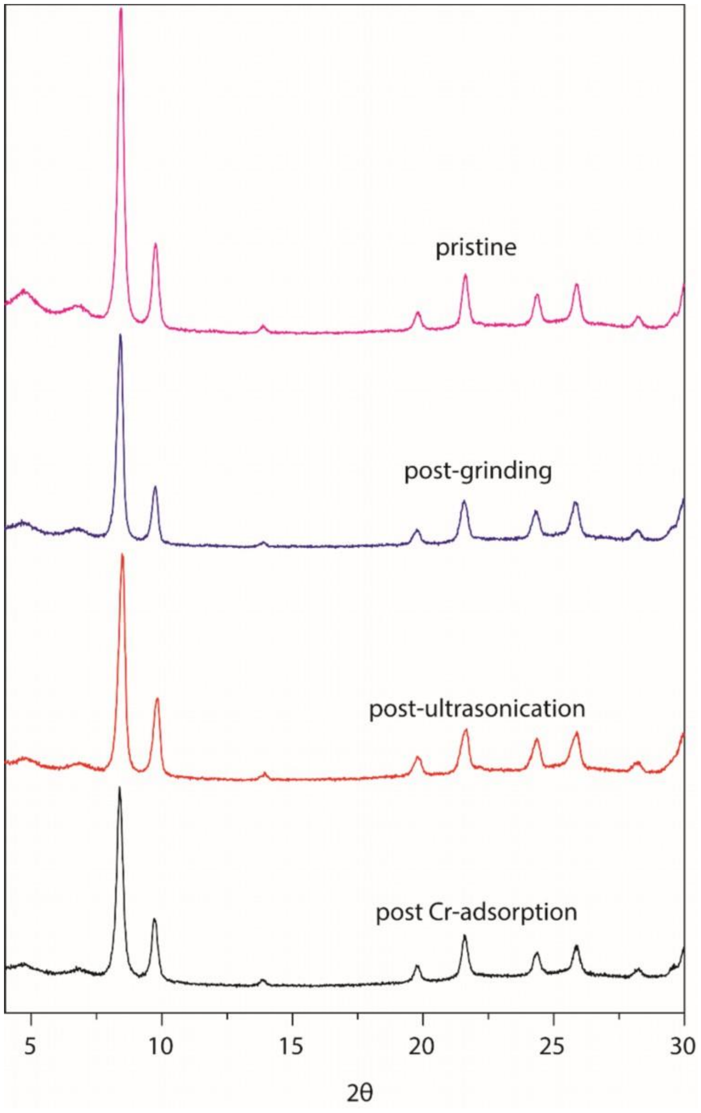

Figure S1 PXRD diffractograms of Zr-MSA after each step of the production and dispersion process.

5. SEM images of 5M's surface before and after Cr adsorption

The surface of 5M samples was analysed before and after the adsorption process. As can be seen in Figure S3, there were no noticeable differences between the two samples, thus confirming the good stability of 5M in water.

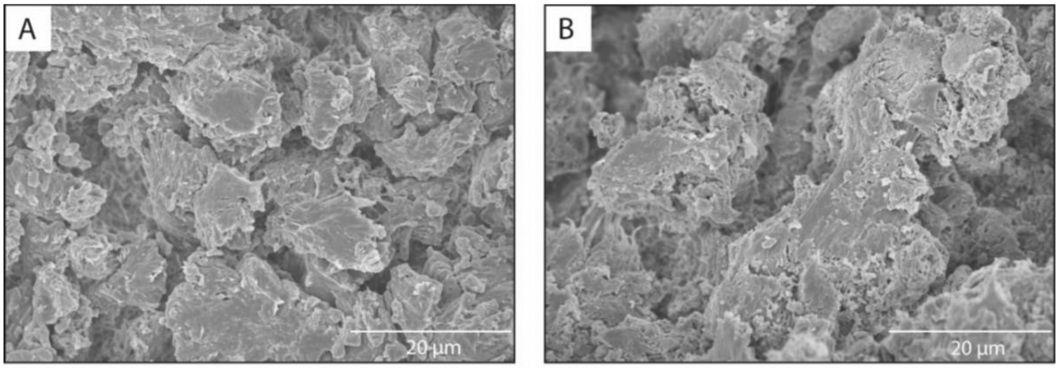

Figure S5 SEM images of 5M membrane A) before and B) after chromium adsorption

6. Colour changes of 5M during Cr(VI) adsorption and reduction

A piece of 5M was visually analysed before its immersion in Cr(VI) solution, after the Cr(VI) adsorption and after the reduction to Cr(III). As can be seen in Figure S4, 5M changed its colour from pale yellow to red to green, depending on the oxidation state of the Cr.

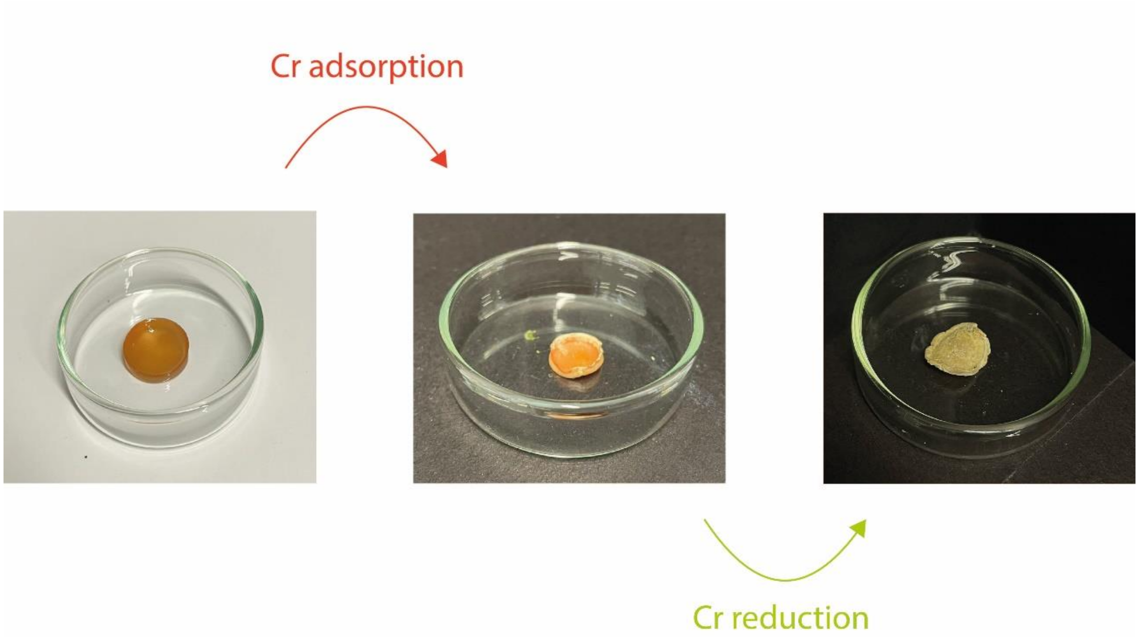

Figure S6 Colour change of 5M during the adsorption and reduction process.

7. EDX analysis of the 5M's surface before the Cr adsorption

The surface of 5M was analysed before the adsorption process in order to map the distribution of the main elements present on the surface. As can be seen, the sulphur distribution was quite homogeneous, with the presence of some denser spot given possibly by the agglomeration of MOFs during the crosslinking process.

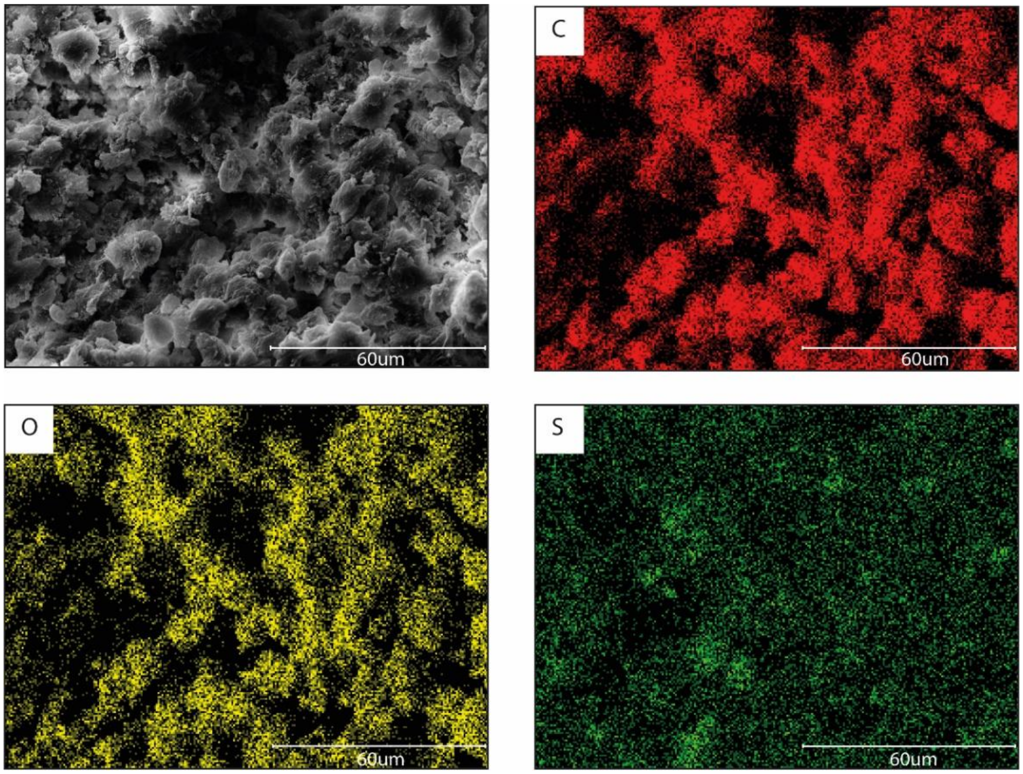

Figure S7 SEM-EDX analysis of the 5M surface before the adsorption process. SEM (upper left) and the mapped elements: carbon (upper right), oxygen (lower left) and sulphur (lower right).

8. Rheological characterization of 5M

Oscillatory strain sweep test and frequency sweep test were performed on 5M. LVR was determined from oscillatory strain sweep test data, shown in Figure S7A. Results showed stable and strain-independent storage and loss moduli up to a strain around 1.3%, a behavior proper of highly structured materials.[1,2] After this point, G' values begun to drop, signaling damage in the gel structure starts, and after 11.4% strain value, the crossover of the moduli took place, at which

point the integrity of the gel was completely lost and the material's liquid-like behavior was predominant. Considering the obtained results, a strain of 1%, in the LVR, was selected for frequency tests. The obtained frequency sweep G' and G'' curves are shown in Figure S6B. It was observed that throughout the whole studied frequency range, constant moduli values were obtained, demonstrating the high stability of the gel that behaved like a highly structured or solid-like material.[3] Moreover, G' values much higher than G'' were measured, proving a predominant elastic behavior.

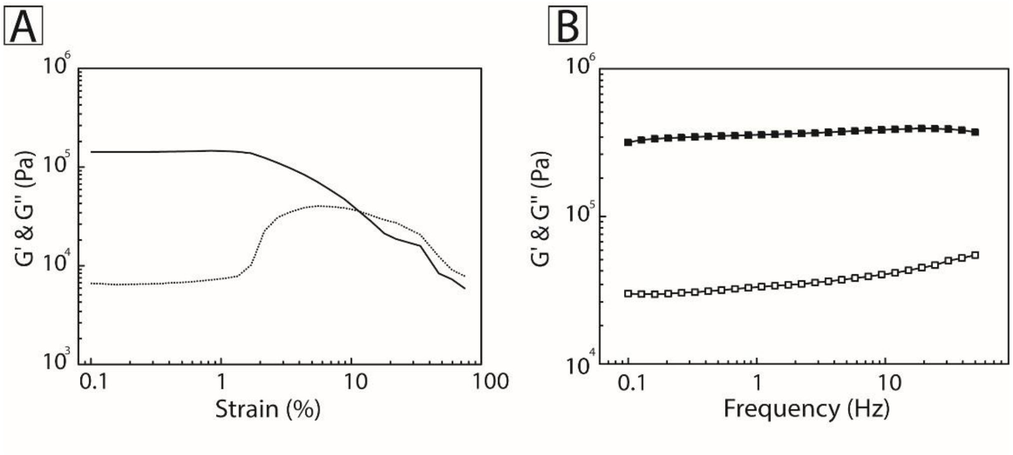

Figure S8 A) Strain amplitude and B) frequency sweep tests curves of 5M gel.

9. Adsorption experiment at higher concentration of Cr(VI)

In this adsorption experiment the adsorbent capacity was set to 2,000 mg L^{-1} while the Cr(VI) concentration was set to 360 mg L^{-1}. The monitored peak was the same at 349 nm, and the absorbance values were collected at 5 min and 1, 6, 24 and 48 h. As can be seen from Figure S7, the main adsorption step occurred during the first hour, when around 72% of the Cr present in the solution was removed. At 6 h, the absorption process was almost complete, with 93% of Cr

removed. The final removal efficiency, after 48 h was estimated to be 99%. The process was followed during 48 hours to verifying the absence of Cr desorption.

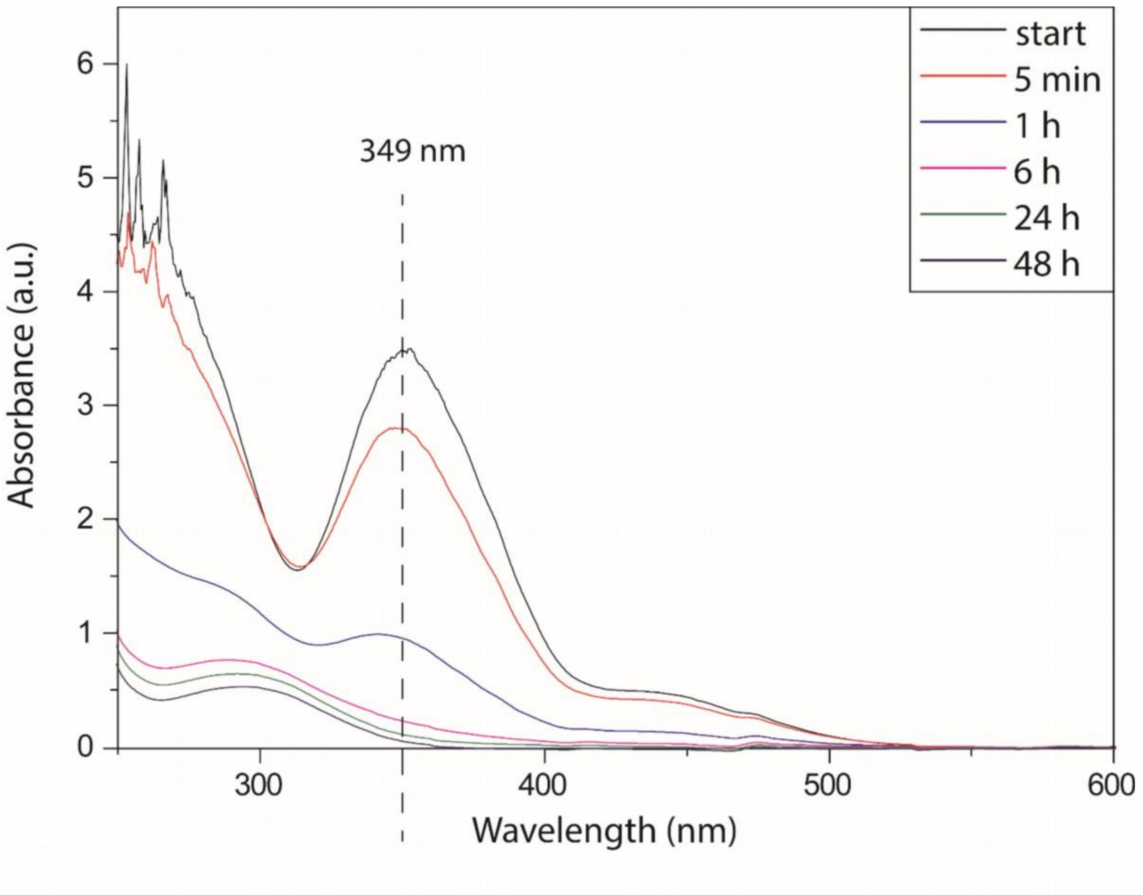

Figure S9 UV-vis absorbance spectra of the Cr(VI) at different times

10. Adsorption kinetics of Zr-MSA powder

As a mean of comparison, the adsorption kinetic of free Zr-MSA powder was studied. To do so, the Zr-MSA concentration was set to 500 mg L^{-1} and that of the Cr(VI) to 130 mg L^{-1}. The same pH and temperature used for 5M were employed. The MOF was simply added to the solution, with

no stirring and the supernatant was analysed using UV-vis, during 5 hours, measuring the changes in the intensity of the peak at 349 nm.

The data were successively fitted using the PSO model, as suggested by other works.[4,5] The k_2 was found to be 0.007 g mg^{-1} min^{-1} while the q_e reached a value of 245.8 mg g^{-1} (R^2= 0.968 and RMSE= 13). The fitted graph is shown in Figure S10.

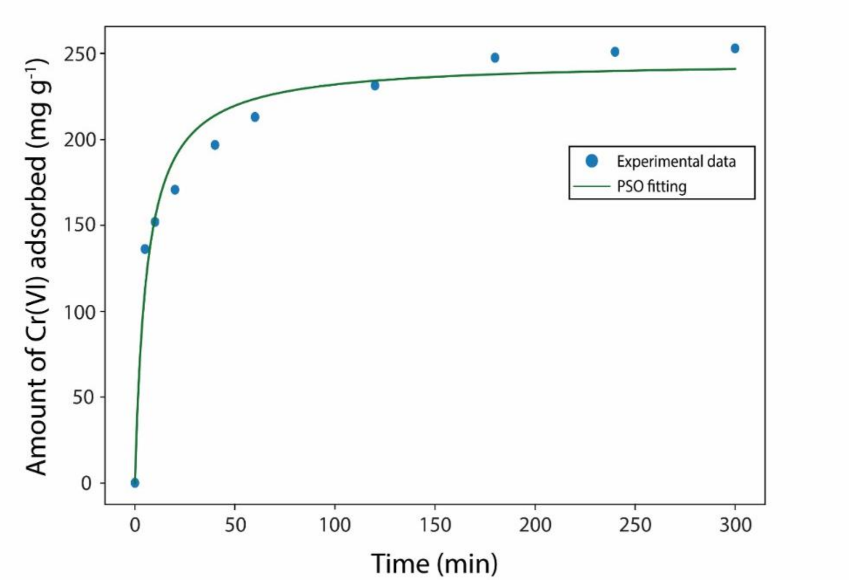

Figure S10 Fitting of the adsorption data recorded for Zr-MSA powder, using the PSO model.

References

[1] R.E. Abouzeid, R. Khiari, D. Beneventi, A. Dufresne, Biomimetic Mineralization of Three-Dimensional Printed Alginate/TEMPO-Oxidized Cellulose Nanofibril Scaffolds for Bone Tissue Engineering, Biomacromolecules 19 (2018) 4442–4452. https://doi.org/10.1021/acs.biomac.8b01325.

[2] J. Vadillo, I. Larraza, T. Calvo-Correas, N. Gabilondo, C. Derail, A. Eceiza, Design of a Waterborne Polyurethane–Urea Ink for Direct Ink Writing 3D Printing, Materials 14 (2021) 3287. https://doi.org/10.3390/ma14123287.
[3] V. Perez-Puyana, M. Jiménez-Rosado, A. Romero, A. Guerrero, Fabrication and Characterization of Hydrogels Based on Gelatinised Collagen with Potential Application in Tissue Engineering, Polymers 12 (2020) 1146. https://doi.org/10.3390/polym12051146.
[4] P. Yang, Y. Shu, Q. Zhuang, Y. Li, J. Gu, Metal–Organic Frameworks Bearing Dense Alkyl Thiol for the Efficient Degradation and Concomitant Removal of Toxic Cr(VI), Langmuir 35 (2019) 16226–16233. https://doi.org/10.1021/acs.langmuir.9b03057.
[5] B. Moll, T. Müller, C. Schlüsener, A. Schmitz, P. Brandt, S. Öztürk, C. Janiak, Modulated synthesis of thiol-functionalized fcu and hcp UiO-66(Zr) for the removal of silver(I) ions from water, Mater. Adv. 2 (2021) 804–812. https://doi.org/10.1039/D0MA00555J.
